# Validity of Diagnostic Codes and Laboratory Tests to Identify Cholangiocarcinoma and Its Subtypes

**DOI:** 10.1002/pds.70154

**Published:** 2025-05-06

**Authors:** Nicole D. Ferrante, Rebecca A. Hubbard, Kelley Weinfurtner, Anya I. Mezina, Craig W. Newcomb, Emma E. Furth, Debika Bhattacharya, Basile Njei, Tamar H. Taddei, Amit Singal, Maarouf A. Hoteit, Lesley S. Park, David Kaplan, Vincent Lo Re

**Affiliations:** ^1^ Division of Gastroenterology and Hepatology, Perelman School of Medicine University of Pennsylvania Philadelphia Pennsylvania USA; ^2^ Center for Real‐World Effectiveness and Safety of Therapeutics, Center for Clinical Epidemiology and Biostatistics, Department of Biostatistics, Epidemiology, and Informatics, Perelman School of Medicine University of Pennsylvania Philadelphia Pennsylvania USA; ^3^ Division of Pathology, Department of Medicine, Perelman School of Medicine University of Pennsylvania Philadelphia Pennsylvania USA; ^4^ Department of Medicine, Division of Infectious Diseases, David Geffen School of Medicine University of California Los Angeles California USA; ^5^ Yale School of Medicine Yale Center for Clinical Investigation New Haven Connecticut USA; ^6^ VA Connecticut Health System West Haven Connecticut USA; ^7^ Department of Medicine Yale University School of Medicine New Haven Connecticut USA; ^8^ Division of Digestive and Liver Diseases UT Southwestern Medical Center Dallas Texas USA; ^9^ Stanford Department of Epidemiology and Population Health Stanford University School of Medicine Palo Alto California USA; ^10^ Department of Medicine Corporal Michael J. Crescenz VA Medical Center Philadelphia Pennsylvania USA; ^11^ Division of Infectious Diseases, Department of Medicine, Perelman School of Medicine University of Pennsylvania Philadelphia Pennsylvania USA

**Keywords:** cholangiocarcinoma, liver cancer, outcomes, validation studies

## Abstract

**Background:**

The absence of validated methods to identify cholangiocarcinoma in real‐world data has prevented the conduct of pharmacoepidemiologic studies to evaluate determinants of this malignancy and examine the effectiveness of cholangiocarcinoma treatments.

**Objective:**

To determine the accuracy of International Classification of Diseases for Oncology, Third Edition (ICD‐O‐3)‐based algorithms to identify cholangiocarcinoma and its subtype (intrahepatic or extrahepatic) within US Veterans Health Administration (VA) data.

**Methods:**

We identified patients with cholangiocarcinoma ICD‐O‐3 diagnosis codes from January 2000–December 2019 in VA data. We developed eight algorithms utilizing ICD‐O‐3 histology codes for cholangiocarcinoma and further used ICD‐O‐3 topography codes for location (liver, intrahepatic bile duct, extrahepatic bile duct) plus maximum total bilirubin (≥ 3 mg/dL vs. < 3 mg/dL) within ± 45 days of diagnosis to identify cholangiocarcinoma subtype. Up to 80 patients were randomly selected for each algorithm, and their records were reviewed by two hepatologists. The positive predictive values (PPV) and 95% confidence interval (CI) for each algorithm were estimated.

**Results:**

Among 2934 unique patients who met inclusion criteria, 574 were randomly selected for validation. All eight algorithms had high PPV for definite or probable cholangiocarcinoma, ranging from 83.8% (95% CI, 73.8%–91.1%) to 100.0% (95% CI, 95.5%–100.0%). Among three algorithms to identify intrahepatic cholangiocarcinoma, two had PPV ≥ 80% (range: 88.8% [95% CI, 79.7%–94.7%]‐91.3% [95% CI, 82.8%–96.4%]). Among five algorithms to identify extrahepatic cholangiocarcinoma, four had PPV ≥ 80% (range: 80.0% [95% CI, 69.6%–88.1%]‐94.0% [83.5%–98.7%]).

**Conclusion:**

These algorithms can be used in future pharmacoepidemiologic studies to evaluate medications associated with intrahepatic or extrahepatic cholangiocarcinoma.

**Summary**

- Electronic healthcare databases are potentially valuable sources for pharmacoepidemiologic studies of cholangiocarcinoma, but algorithms to identify cholangiocarcinoma diagnoses and ascertain its subtype as intrahepatic or extrahepatic have not been developed and validated within US healthcare data.
- We developed eight algorithms based on International Classification of Diseases for Oncology, Third Edition (ICD‐O‐3) histology (cholangiocarcinoma, Klatskin tumor, adenocarcinoma) and topography (liver, intrahepatic bile duct, extrahepatic bile duct) codes, in combination with maximum total bilirubin levels for select algorithms, to identify cholangiocarcinoma diagnoses and their subtype.
- All eight ICD‐O‐3‐based algorithms had a positive predictive value (PPV) of at least 83.8% (range: 83.8%–100.0%) for hepatologist‐confirmed definite or probable cholangiocarcinoma.
- Two of the three algorithms created to identify intrahepatic cholangiocarcinoma had high PPV for definite or probable intrahepatic cholangiocarcinoma, ranging from 88.8% to 91.3%. Four of the five algorithms created to identify extrahepatic cholangiocarcinoma had high PPV for definite or probable extrahepatic cholangiocarcinoma, ranging from 80.0% to 94.0%.
- These algorithms could be used in future pharmacoepidemiologic studies to evaluate medications associated with intrahepatic or extrahepatic cholangiocarcinoma within US Veterans Health Administration data.

## 1 Introduction

Cholangiocarcinoma is an aggressive malignancy that arises from bile duct epithelial cells [1, 2]. It is the second most common hepatic malignancy after hepatocellular carcinoma (HCC), representing 10%–15% of primary liver cancers and 3% of all gastrointestinal cancers [3]. Moreover, there are three subtypes of cholangiocarcinoma defined by the anatomic site of origin within the biliary tree: (1) intrahepatic cholangiocarcinoma, arising above the second‐order branches of the bile ducts; (2) perihilar cholangiocarcinoma, which develops below the second‐order branches of the bile ducts and above the insertion of the cystic duct; and (3) distal cholangiocarcinoma, which arises below the insertion of the cystic duct [3]. Collectively, perihilar and distal cholangiocarcinoma are typically referred to as extrahepatic cholangiocarcinoma.

Over the last three decades, the incidence of cholangiocarcinoma has been increasing worldwide [4, 5, 6], likely due to the rising incidence of metabolic syndrome [4, 6, 7, 8, 9]. Yet, major knowledge gaps remain on the determinants and comparative effectiveness of medical therapies of cholangiocarcinoma and its subtypes, largely because methods to validly identify this malignancy within real‐world data have been lacking. Electronic health record (EHR) databases could be valuable resources for studying the pharmacoepidemiology of cholangiocarcinoma and its subtypes. However, methods to identify cholangiocarcinoma cases and ascertain their subtype must first be developed and validated.

To address this methodologic need, we developed and evaluated the performance of eight case‐finding algorithms for cholangiocarcinoma and its subtypes using cancer registry coded (International Classification of Diseases for Oncology, Third Edition [ICD‐O‐3]) diagnoses [10], alone or in combination with total bilirubin values, within EHR data of the US Veterans Health Administration (VA). Eight algorithms were developed based on unique combinations of ICD‐O‐3 histology and topography codes, alone or in combination with total bilirubin levels. Specifically, we evaluated the positive predictive value (PPV) of algorithms to: (1) identify cholangiocarcinoma cases, and (2) classify cholangiocarcinoma cases by anatomic subtype as intrahepatic or extrahepatic.

## 2 Methods

### 2.1 Design and Data Source

We conducted a retrospective study using EHR data from the national VA system between January 1, 2000 and December 31, 2019. The VA Corporate Data Warehouse (CDW) is a national, continually updated repository of data extracted from the VA's EHR for all clinical encounters across all VA sites in the US [11, 12]. The VA CDW contains data on enrollment, demographics, medical diagnoses recorded using International Classification of Diseases, Ninth and Tenth Revision (ICD‐9/−10) diagnoses, procedures (recorded using Current Procedural Terminology [CPT] codes), and dispensed medications.

VA EHR data can be linked to the VA national cancer registry, which collects records on all cancers diagnosed and/or treated within the VA system [13, 14, 15]. Information on cancer diagnosis and treatment is compiled and submitted by local cancer registrars at each of the VA medical centers that diagnose and/or manage veterans with cancer. The information that is aggregated by the cancer registrars is encoded to meet site‐specific requirements for registry inclusion, as established by several oversight bodies [14, 15]. The information obtained by the cancer registrars is obtained from the medical records for each patient and includes extensive information about demographics, method of cancer identification, histopathology, tumor stage, treatments, and date of recurrence. All cancers recorded in the VA national cancer registry can be identified using ICD‐O‐3 codes, specifically, one ICD‐O‐3 histology code (to classify the tissue histopathology) in combination with one ICD‐O‐3 topography code (to classify the primary location of the cancer) [10]. This study was approved by the Institutional Review Boards of the Corporal Michael J. Crescenz Philadelphia VA Medical Center, VA Connecticut Healthcare System, and Yale University and deemed exempt by the University of Pennsylvania with a waiver of informed consent.

### 2.2 Patients Selected for Validation

We first identified ICD‐O‐3 histology and topography codes that might represent a cholangiocarcinoma diagnosis (Table S1). Because cholangiocarcinoma can have several histological features, with adenocarcinoma being the most common, we explored several histology codes (Table S1). We further evaluated the frequency of use of each ICD‐O‐3 code.

Initially, we developed seven algorithms with the potential to identify cholangiocarcinoma and its location (extrahepatic or intrahepatic) based on unique combinations of ICD‐O‐3 histology and topography codes. Preliminarily, one of our algorithms (cholangiocarcinoma histology code [8160] + intrahepatic bile duct topography code [C22.1]) appeared to perform poorly for differentiating cholangiocarcinoma subtype. To enhance the performance of our algorithm, we incorporated a maximum total bilirubin (Tbili) cutoff of ≥ 3 vs. < 3 mg/dL within ± 45 days of the cancer diagnosis date. This decision was based on the presence or absence of biliary obstruction potentially helping to differentiate between intrahepatic and extrahepatic cholangiocarcinoma, such that extrahepatic cholangiocarcinoma is often associated with greater degrees of biliary obstruction and higher Tbili levels. A Tbili cutoff of 3 mg/dL was chosen because patients with biliary obstruction typically develop a Tbili ≥ 3 mg/dL, and this threshold signifies the level at which clinically apparent jaundice occurs. A total of eight algorithms were evaluated. Although the bile duct cystadenoma ICD‐O‐3 histology code (8163) can identify cholangiocarcinoma, it was not included since only one patient had this code recorded during the period under observation.

For each algorithm, we randomly selected 80 potential cholangiocarcinoma cases who had complete data abstracted by the cancer registrar, a diagnosis that was confirmed based on cytology or histology results, and either carcinoma in situ (cancer behavior = 2) or malignancy (cancer behavior = 3). If there were less than 80 potential events for any algorithm, then all patients were selected who had complete abstraction by the registrar and either carcinoma in situ or malignancy, regardless of their method of confirmation. An alternative patient was randomly identified if the initially sampled patient had incomplete data to ensure at least 80 patients were adjudicated. The algorithms were constructed as follows:

-
  Algorithm 1 *Cholangiocarcinoma histology code* (8160) *+ liver topography code* (C22.0).
-
  Algorithm 2 *Cholangiocarcinoma histology code* (8160) *+ intrahepatic bile duct topography code* (C22.1) *+ maximum Tbili ≥* 3 *mg/dL within ±* 45 *days of cancer diagnosis date*.
-
  Algorithm 3 *Cholangiocarcinoma histology code* (8160) *+ intrahepatic bile duct topography code* (C22.1) *+ maximum Tbili <* 3 *mg/dL within ±* 45 *days of cancer diagnosis date*.
-
  Algorithm 4 *Klatskin tumor histology code* (8162) *+ liver* (C22.0) *or intrahepatic bile duct topography code* (C22.1).
-
  Algorithm 5 *Cholangiocarcinoma histology code* (8160) *+ extrahepatic bile duct* (C24.0) *topography code*.
-
  Algorithm 6 *Klatskin tumor histology code* (8162) *+ extrahepatic bile duct* (C24.0) *topography code*.
-
  Algorithm 7 *Adenocarcinoma not otherwise specified (NOS) histology code* (8140) *+ intrahepatic bile duct topography code* (C22.1).
-
  Algorithm 8 *Adenocarcinoma NOS histology code* (8140) *+ extrahepatic bile duct topography code* (C24.0).

### 2.3 Definitions of Cholangiocarcinoma and Subtypes

#### 2.3.1 Cholangiocarcinoma Case Definition

A cholangiocarcinoma diagnosis was confirmed if the patient met the case definition for a definite or probable event (Table 1). A definite diagnosis was based on the National Comprehensive Cancer Network Clinical Practice Guidelines in Oncology Version 1.2022 and the 2019 World Health Organization Classification of Tumors of the Digestive System definition for cholangiocarcinoma (Table 1) [16, 17]. A probable diagnosis was based on the presence of a presumed malignant dominant biliary stricture or hepatobiliary mass lesion on cross‐sectional imaging, endoscopic retrograde cholangiopancreatography (ERCP), or endoscopic ultrasound (EUS) in the absence of another primary lesion (i.e., absence of a pancreatic head mass, HCC, or other primary) plus at least 1 minor criterion (Table 1). The probable diagnosis was included because a histopathologic diagnosis can be difficult to confirm in clinical practice and since a preoperative biopsy is not always clinically necessary prior to definitive therapy. We classified cholangiocarcinoma as absent if there was no evidence of cancer. Cholangiocarcinoma was considered indeterminate if its presence could not be confirmed with the data available.

**TABLE 1:** Criteria for defining definite and probable cholangiocarcinoma and subtypes.

Definite cholangiocarcinoma	Probable cholangiocarcinoma
ONE of the following: Cholangiocarcinoma on histocytopathology Adenocarcinoma on histocytopathology that favors cholangiocarcinoma or pancreaticobiliary source based on immunohistochemical studies in the absence of a pancreatic mass Bile duct biopsy/brushings that demonstrates adenocarcinoma in the absence of a pancreatic mass FISH consistent with cholangiocarcinoma (polysomy or aneuploidy) in the presence of a dominant biliary stricture (bile duct brushings)	BOTH of the following: Presumed malignant hepatobiliary mass lesion or dominant biliary stricture on imaging (CT, MRI, ERCP, or EUS) Low suspicion for another primary site of cancer, including the absence of a pancreatic mass on cross‐sectional imaging or EUS and low suspicion for HCC AND at least one of the following minor criteria: Elevated tumor markers (CA 19–9 > 100 U/mL) Histocytopathology with “malignant cells” or that is “suspicious for adenocarcinoma” or “suspicious for cholangiocarcinoma” Liver biopsy that demonstrates adenocarcinoma in the setting of a hilar mass, solitary hepatic mass, or dominant liver lesion^a^ Multidisciplinary consensus of cholangiocarcinoma by ≥ 2 subspecialties or tumor board review.

Definite intrahepatic cholangiocarcinoma	Probable intrahepatic cholangiocarcinoma
Hepatic mass lesion(s) in the absence of a biliary stricture involving the biliary tree below the second order bile ducts (i.e., the biliary stricture does not involve the right or left intrahepatic duct, bifurcation of the ducts, hilum, common hepatic duct, or common bile duct)	Perihilar mass lesion in the absence of a biliary stricture below the second order bile ducts and absence of biliary obstruction^b^

Definite extrahepatic cholangiocarcinoma	Probable extrahepatic cholangiocarcinoma
ONE of the following, with or without concomitant liver lesion(s) concerning for metastatic disease: Biliary stricture arising below the second order bile ducts (involving the right or left intrahepatic duct, bifurcation of the ducts, hilum, common hepatic duct, or common bile duct) on imaging or ERCP (Bismuth‐Corlette Types I, II, IIIa, IIIb, IV, V)	Biliary stricture or mass lesion arising below the second order bile ducts on imaging or ERCP with atypical cells on bile duct brushings

Abbreviations: CA 19–9 = carbohydrate antigen 19–9, CT = computed tomography, ERCP = endoscopic retrograde cholangiopancreatography, EUS = endoscopic ultrasound, FISH = fluorescence in situ hybridization, GI = gastrointestinal, HCC = hepatocellular carcinoma, MRI = magnetic resonance imaging.

^a^ Dominant liver lesion: In the setting of multifocal liver lesions, a dominant liver lesion is a lesion that is at least 4 times the diameter of the next largest lesion.

^b^ Biliary obstruction: Dilation of a bile duct associated with an elevation in total bilirubin ≥ 2 mg/dL.

#### 2.3.2 Cholangiocarcinoma Subtype Definition

Cholangiocarcinoma subtype was ascertained using National Comprehensive Cancer Network Clinical Practice Guidelines in Oncology Version 1.2022 [16]. Cholangiocarcinoma subtype was confirmed if the patient met the clinical case definition for definite or probable intrahepatic or extrahepatic cholangiocarcinoma (Table 1). The probable subtype definition was included because ascertaining the location can be challenging in clinical practice and requires review with a radiologist or multidisciplinary team. This most commonly occurs when there is a mass in the hilar region, but there is uncertainty as to whether the mass originates from the liver parenchyma or hilum. The presence or absence of biliary obstruction can help to differentiate between intrahepatic and extrahepatic cholangiocarcinoma.

### 2.4 Confirmation of Cholangiocarcinoma Case and Subtype

A single trained data abstractor reviewed the medical records of all patients selected for validation. The following data were abstracted into structured forms using Research Electronic Data Capture (REDCap) tools: (1) pathology reports, including histopathology, cytology, surgical pathology, and autopsy reports (to confirm cholangiocarcinoma diagnosis and ascertain subtype); (2) laboratory test results, including total bilirubin, cancer antigen (CA) 19–9, and alpha fetoprotein (to support the cholangiocarcinoma diagnosis and its location); (3) imaging and procedure reports, including computed tomography (CT), magnetic resonance imaging (MRI), ERCP, and EUS reports, as well as esophagogastroduodenoscopy and colonoscopy reports in select cases (to confirm cholangiocarcinoma diagnosis, ascertain location, and exclude other primary cancer sites); and (4) progress notes from oncology and gastroenterology consultants, operative reports, and tumor board notes (to confirm cholangiocarcinoma diagnosis). The diagnosis date was obtained from cancer registry data.

Abstraction forms were independently reviewed by two hepatologists who classified diagnoses as definite, probable, indeterminate, or absent. The same two hepatologists classified the anatomic subtype as definite intrahepatic, probable intrahepatic, definite extrahepatic, probable extrahepatic, indeterminate subtype, or not applicable (for patients who did not have a cholangiocarcinoma diagnosis confirmed). For patients adjudicated as having definite or probable intrahepatic or extrahepatic cholangiocarcinoma but indeterminate for cholangiocarcinoma status, they were treated as indeterminate for subtype. Any disagreement in diagnosis or subtype resulted in review by a third hepatologist to arbitrate the case.

### 2.5 Statistical Analyses

We calculated the positive predictive value (PPV) with exact binomial 95% confidence interval (CI) of each algorithm for confirmed definite or probable cholangiocarcinoma. We focused on PPV because a sufficiently high PPV will provide confidence that identified outcomes represent true events. Because cholangiocarcinoma is rare, we expected that the false‐negative probability of our algorithms would be very low and, correspondingly, the negative predictive value was expected to be very high. A cholangiocarcinoma case‐finding algorithm with high PPV will have low contamination with false‐positive cases. Since probable events are very likely to represent cholangiocarcinoma diagnoses, we felt that including these in the calculation for PPV was appropriate.

Assuming a PPV of 80%, we estimated that a sample of 80 patients for each algorithm would allow estimation of the PPV with a 95% CI width of ± 10%, which was deemed sufficiently narrow. We measured interrater agreement between the two reviewers for cholangiocarcinoma diagnosis and subtype by calculating the percent agreement and kappa statistic for cholangiocarcinoma diagnosis and subtype, both overall and separately within each subsample identified for validation of each algorithm. Next, we calculated the PPV with 95% CI for each algorithm for confirmed definite or probable cholangiocarcinoma subtype, i.e., intrahepatic or extrahepatic cholangiocarcinoma. We then calculated the combined PPV with 95% CI for intrahepatic and extrahepatic cholangiocarcinoma using the algorithms that performed well for each subtype. In a secondary analysis, we determined the PPV with 95% CI for each algorithm for definite (only) cholangiocarcinoma, as well as definite (only) intrahepatic or definite (only) extrahepatic cholangiocarcinoma. We also explored reasons for indeterminate cholangiocarcinoma status.

## 3 Results

### 3.1 Patients Selected for Validation

Between January 1, 2000, and December 31, 2019, we identified 2934 unique patients who met the criteria of one of our eight algorithms (Algorithm 1: 493, 2: 340, 3: 663, 4: 44, 5: 712, 6: 51, 7: 137, 8: 494). No patients were assigned to more than one algorithm. Among the 574 patients who were randomly selected for validation, 13 were resampled due to the lack of availability of data in the medical record. All patients with complete data were adjudicated.

### 3.2 Performance of Algorithms for Cholangiocarcinoma

Among the 574 patients selected for validation, 394 (68.6%) were adjudicated as having definite cholangiocarcinoma, 141 (24.6%) as having probable cholangiocarcinoma, 30 (5.2%) as being indeterminate, and 9 (1.6%) were classified as not having cholangiocarcinoma. Overall, there was high agreement between the two reviewers for cholangiocarcinoma status (percent agreement, 92.2%; kappa‐statistic, 0.85). All eight algorithms had high PPV for confirmed definite or probable cholangiocarcinoma, ranging between 83.8%–100.0% (Table 2). Algorithms that incorporated a cholangiocarcinoma or Klatskin tumor histology code had at least 90.9% PPV for definite or probable cholangiocarcinoma: Algorithm 1: PPV = 100.0% (95% CI, 95.5%–100.0%); Algorithm 2: PPV = 95.0% (95% CI, 87.7%–98.6%); Algorithm 3: PPV = 92.5% (95% CI, 84.4%–97.2%); Algorithm 4: PPV = 90.9% (95% CI, 78.3%–97.5%); Algorithm 5: PPV = 95.0% (95% CI, 87.7%–98.6%); Algorithm 6: PPV = 94.0% (95% CI, 83.5%–98.7%). Algorithms that incorporated an adenocarcinoma NOS histology code also had high PPV for confirmed definite or probable cholangiocarcinoma: Algorithm 7: PPV = 93.8% (95% CI, 86.0%–97.9%); Algorithm 8: PPV = 83.8% (95% CI, 73.8%–91.1%). The most common reasons that patients were not adjudicated as having definite or probable cholangiocarcinoma were: 1) misclassified or unknown tumor site, or 2) lack of diagnostic confirmation (Table 3).

**TABLE 2:** Positive predictive values with 95% confidence intervals (CI) of case‐identifying algorithms for cholangiocarcinoma events.

Alg.#	ICD‐O‐3 histology code + topography code combination			Laboratory test	*N* meeting algorithm	*N* sampled	*N* confirmed CCA^a^	PPV for definite or probable CCA (95% CI)	PPV for definite CCA (95% CI)	Kappa statistic
	ICD‐O‐3 histology code		ICD‐O‐3 topography code							
1	Cholangiocarcinoma (8160)	+	Liver (C22.0)		493	80	80	100% (95.5%–100.0%)	91.3 (82.8%–96.4%)	1.00
2	Cholangiocarcinoma (8160)	+	Intrahepatic bile duct (C22.1)	+ Tbili ≥ 3 mg/dL^b^	340	80	76	95.0% (87.7%–98.6%)	65.0 (53.5%–75.3%)	0.90
3	Cholangiocarcinoma (8160)	+	Intrahepatic bile duct (C22.1)	+ Tbili < 3 mg/dL^b^	663	80	74	92.5% (84.4%–97.2%)	70.0 (58.7%–79.7%)	0.89
4	Klatskin tumor (8162)	+	Liver (C22.0) or intrahepatic bile duct (C22.1)		44	44	40	90.9% (78.3%–97.5%)	34.1 (20.5%–49.9%)	0.84
5	Cholangiocarcinoma (8160)	+	Extrahepatic bile duct (C24.0)		712	80	76	95.0% (87.7%–98.6%)	80.0 (69.6%–88.1%)	0.84
6	Klatskin tumor (8162)	+	Extrahepatic bile duct (C24.0)		51	50	47	94.0% (83.5%–98.7%)	44.0 (30.0%–58.7%)	0.83
7	Adenocarcinoma NOS (8140)	+	Intrahepatic bile duct (C22.1)		137	80	75	93.8% (86.0%–97.9%)	70.0 (58.7%–79.7%)	0.82
8	Adenocarcinoma NOS (8140)	+	Extrahepatic bile duct (C24.0)		494	80	67	83.8% (73.8%–91.1%)	70.0 (58.7%–79.7%)	0.72

Abbreviations: CCA = cholangiocarcinoma, ICD‐O‐3 = International Classification of Diseases for Oncology, Third Edition, PPV = positive predictive value, Tbili = total bilirubin level.

^a^ Confirmed cholangiocarcinoma based on definite or probable diagnoses.

^b^ Within ± 45 days of the cancer diagnosis date.

**TABLE 3:** Reasons why patients selected for validation did not have confirmed definite or probable cholangiocarcinoma.

Alg.#	ICD‐O‐3 histology code + topography code combination		*N* sampled				Reason not confirmed CCA			
	ICD‐O‐3 histology code		ICD‐O‐3 topography code	*N* confirmed CCA^a^	*N* without confirmed CCA		Gallbladder cancer	Pancreatic cancer	Other primary	Other
1	Cholangiocarcinoma (8160)	+	Liver (C22.0)	80	80	0	—	—	—	—
2	Cholangiocarcinoma (8160)	+	Intrahepatic bile duct (C22.1) + Tbili ≥ 3 mg/dL^b^	80	76	4	—	—	3 (1 Thyroid, 2 HCC)	1 Unknown primary
3	Cholangiocarcinoma (8160)	+	Intrahepatic bile duct (C22.1) + Tbili < 3 mg/dL^b^	80	74	6	1	2	1 (Colorectal)	1 Unknown primary
4	Klatskin tumor (8162)	+	Liver (C22.0) or intrahepatic bile duct (C22.1)	44	40	4	1	—	—	3 (1 Nondiagnostic biliary sample; 2 without tissue sample or labs)
5	Cholangiocarcinoma (8160)	+	Extrahepatic bile duct (C24.0)	80	76	4	—	—	1 (lung)	1 (Nondiagnostic biliary samples)
6	Klatskin tumor (8162)	+	Extrahepatic bile duct (C24.0) or gallbladder (C23.9)	50	47	3	—	1	—	2 (Nondiagnostic biliary samples)
7	Adenocarcinoma NOS (8140)	+	Intrahepatic bile duct topography (C22.1)	80	75	5	3	1	1 (lung)	—
8	Adenocarcinoma NOS (8140)	+	Extrahepatic bile duct topography (C24.0)	80	67	13	—	10	2 (ampullary)	1 Without contrast enhanced imaging so could not rule out other primary

Abbreviations: CCA = cholangiocarcinoma, ICD‐O‐3 = International Classification of Diseases for Oncology, Third Edition, Tbili = total bilirubin level.

^a^ Confirmed cholangiocarcinoma based on definite or probable diagnoses.

^b^ Within ± 45 days of the cancer diagnosis date.

### 3.3 Performance of Algorithms for Cholangiocarcinoma Subtype

Among the 574 patients selected for validation, 319 (55.6%) were adjudicated as having definite extrahepatic cholangiocarcinoma, 197 (34.3%) as having definite intrahepatic cholangiocarcinoma, 26 (4.5%) as having probable intrahepatic cholangiocarcinoma, 6 (1.0%) as having probable extrahepatic cholangiocarcinoma, and 26 (4.5%) had an indeterminate subtype. Overall, there was substantial agreement between the two reviewers for cholangiocarcinoma subtype (percent agreement, 87.1%; kappa‐statistic, 0.78).

The interrater reliability and PPV of each algorithm to identify cholangiocarcinoma subtype are shown in Table 4. Algorithms 1 and 3 had high PPV for confirmed definite or probable intrahepatic cholangiocarcinoma with 91.3% PPV (95% CI, 82.8%–96.4%) and 88.8% PPV (95% CI, 79.7%–94.7%), respectively, and a combined PPV of 90.0% (95% CI, 84.3%–94.2%). In contrast, algorithm 7 had low PPV for definite or probable intrahepatic cholangiocarcinoma (PPV, 45.0%; 95% CI, 33.8%–56.5%). Algorithms 4, 5, 6, and 8 had high PPV for confirmed definite or probable extrahepatic cholangiocarcinoma with 88.6% PPV (95% CI, 75.4%–96.2%), 85.0% PPV (95% CI, 75.3%–92.0%), 94.0% PPV (95% CI, 83.5%–98.7%), and 80.0% PPV (95% CI, 69.6%–88.1%), respectively, and a combined PPV of 85.8% (95% CI, 80.9%–89.9%). In contrast, Algorithm 2 had marginal PPV for definite or probable extrahepatic cholangiocarcinoma with 72.5% PPV (95% CI, 61.4%–81.9%).

**TABLE 4:** Positive predictive value (95% confidence interval) of case‐identifying algorithms for intrahepatic and extrahepatic cholangiocarcinoma.

Alg. #	ICD‐O‐3 code histology + topography code combination			*N* meeting algorithm	*N* selected for validation	*N* confirmed definite or probable intrahepatic or extrahepatic CCA	PPV for definite or probable intrahepatic or extrahepatic CCA (95% CI)	PPV for definite intrahepatic or extrahepatic CCA (95% CI)	Kappa statistic
	ICD‐O‐3 histology code		ICD‐O‐3 topography code						
Specified Location									
Intrahepatic									
1	Cholangiocarcinoma (8160)	+	Liver (C22.0)	493	80	73	91.3% (82.8%–96.4%)	86.3 (76.7%–92.9%)	0.73
3	Cholangiocarcinoma (8160)	+	Intrahepatic bile duct (C22.1) + max Tbili < 3^c^	663	80	71	88.8 (79.7%–94.7%)	85.0 (75.3%–92.0%)	0.81
7	Adenocarcinoma NOS (8140)	+	Intrahepatic bile duct topography (C22.1)	137	80	36	45.0 (33.8%–56.5%)	41.3 (30.4%–52.8%)	0.61
1, 3^a^	Algorithm 1 or Algorithm 3			1156	160	144	90.0 (84.3%–94.2%)	85.6 (79.2%–90.7%)	
Extrahepatic									
2	Cholangiocarcinoma (8160)	+	Intrahepatic bile duct (C22.1) + max Tbili > = 3^c^	340	80	58	72.5 (61.4%–81.9%)	70.0 (58.7%–79.7%)	0.79
4	Klatskin tumor (8162)	+	Liver (C22.0) or intrahepatic bile duct (C22.1)	44	44	39	88.6 (75.4%–96.2%)	84.1 (69.9%–93.4%)	0.29
5	Cholangiocarcinoma (8160)	+	Extrahepatic bile duct (C24.0)	712	80	68	85.0 (75.3%–92.0%)	85.0 (75.3%–92.0%)	0.86
6	Klatskin tumor (8162)	+	Extrahepatic bile duct (C24.0)	51	50	47	94.0 (83.5%–98.7%)	92.0 (80.8%–97.8%)	0.33
8	Adenocarcinoma NOS (8140)	+	Extrahepatic bile duct (C24.0)	494	80	64	80.0 (69.6%–88.1%)	78.8 (68.2%–87.1%)	0.33
4, 5, 6, 8^b^	Algorithm 4, 5, 6, or 8			1301	254	218	85.8 (80.9%–89.9%)	84.3 (79.2%–88.5%)	

Abbreviations: CCA = cholangiocarcinoma, ICD‐O‐3 = International Classification of Diseases for Oncology, Third Edition, PPV = positive predictive value, Tbili = total bilirubin level.

^a^ The combined PPV for definite or probable intrahepatic CCA was determined by combining the algorithms with > 80% positive predictive value for intrahepatic CCA. Patients had to meet the criteria for Algorithms 1 or 3 (cholangiocarcinoma histology code [8160] + liver topography code [C22.0] or intrahepatic bile duct topography code [C22.1] + maximum Tbili < 3 mg/dL).

^b^ The combined PPV for definite or probable extrahepatic CCA was determined by combining the algorithms with > 80% positive predictive value for extrahepatic CCA. Patients had to meet the criteria for Algorithms 4, 5, 6, or 8 (Klatskin tumor histology code [8162] + liver [C22.0) or intrahepatic bile duct [C22.1] or extrahepatic bile duct topography code [C24.0] or cholangiocarcinoma histology code [8160] + extrahepatic bile duct topography code [C24.0] or adenocarcinoma NOS histology code [8140] + extrahepatic bile duct topography code [C24.0]).

^c^ Within ± 45 days of the cancer diagnosis date.

## 4 Discussion

We found that all eight algorithms that we developed had a high PPV for confirmed definite or probable cholangiocarcinoma, with PPVs ranging from 83.8%–100.0%. Six of the eight algorithms had a high PPV for cholangiocarcinoma subtype. Among three algorithms created to identify intrahepatic cholangiocarcinoma, two had PPV ≥ 80%. Among 5 algorithms created to identify extrahepatic cholangiocarcinoma, four had a PPV ≥ 80%. Algorithms that utilized an adenocarcinoma NOS histology code identified cholangiocarcinoma cases with high PPV but were less reliable for identifying cholangiocarcinoma subtype.

This work is important for future pharmacoepidemiologic research on cholangiocarcinoma. VA EHR data could allow for the evaluation of medications associated with cholangiocarcinoma and its subtypes in large cohorts. Moreover, while there is an increasing number of therapies being utilized to treat cholangiocarcinoma, such as liver transplantation, locoregional therapy, chemotherapy, and targeted immunotherapy, their comparative effectiveness at the population level has not been fully elucidated. These, too, could be evaluated within VA EHR data. Future studies could also help elucidate other determinants of cholangiocarcinoma subtypes, which remain major knowledge gaps in cholangiocarcinoma research.

Previous epidemiologic studies of cholangiocarcinoma have utilized administrative databases to analyze trends, ascertain risk factors, and identify disparities in access to care for cholangiocarcinoma, but these relied on unvalidated ICD‐9/10 codes to identify cholangiocarcinoma and its subtypes. A US retrospective cohort study performed by Welzel et al. [18] examining the National Cancer Institute's Surveillance, Epidemiology, and End Results cancer registry found that 91% of perihilar cholangiocarcinoma diagnoses were incorrectly coded as intrahepatic cholangiocarcinoma, resulting in an overestimation of intrahepatic cholangiocarcinoma incidence. Similarly, a UK study reviewing 625 hepatobiliary malignancies from three centers found that only 43% of cholangiocarcinoma cases coded as intrahepatic cholangiocarcinoma using ICD‐10 codes were true intrahepatic cholangiocarcinoma cases and that 34% of cholangiocarcinomas coded as intrahepatic cholangiocarcinoma were perihilar [19]. Our use of maximum Tbili level to augment ICD coding should increase specificity compared to prior studies.

Since cholangiocarcinoma can have different histological features based on the World Health Organization classification of its histopathology, we also explored several different ICD‐O‐3 histology codes, including adenocarcinoma NOS (ICD‐O‐3 code 8140) and carcinoma NOS (ICD‐O‐3 code 8010). Among these exploratory ICD‐O‐3 histology codes, adenocarcinoma NOS (ICD‐O‐3 code 8140) yielded the greatest number of potential cholangiocarcinoma events, so it was incorporated within two algorithms, one in combination with an intrahepatic bile duct topography code to identify intrahepatic cholangiocarcinoma cases (Algorithm 7) and one with an extrahepatic bile duct topography code to identify extrahepatic cholangiocarcinoma cases (Algorithm 8). We did not include an algorithm consisting of an adenocarcinoma NOS (ICD‐O code 8140) histology code plus a liver (ICD‐10 C22.1) topography code because this combination would be challenging to differentiate true cholangiocarcinoma events from metastatic adenocarcinoma.

Of the three algorithms developed to identify intrahepatic cholangiocarcinoma (Algorithms 1, 3, and 7), Algorithms 1 and 3 had high PPV for confirmed definite or probable intrahepatic cholangiocarcinoma. Algorithm 7 identified 138 patients with an adenocarcinoma NOS histology and intrahepatic bile duct topography but was unable to ascertain cholangiocarcinoma subtype, as 45.0% (36/80) had intrahepatic cholangiocarcinoma and 48.8% (39/80) had extrahepatic cholangiocarcinoma. Of the five algorithms developed to identify extrahepatic cholangiocarcinoma (Algorithms 2, 4, 5, 6, and 8), Algorithms 4, 5, 6, and 8 had high PPV for confirmed definite or probable extrahepatic cholangiocarcinoma. While Algorithm 2 identified 340 patients with a cholangiocarcinoma histology code, extrahepatic bile duct topography code, and maximum Tbili ≥ 3 mg/dL within ± 45 days of the cancer diagnosis date, it had poor performance, with 72.5% PPV for confirmed extrahepatic cholangiocarcinoma. We suspect that this algorithm performed poorly because it captured patients with intrahepatic cholangiocarcinoma who had hyperbilirubinemia, such as patients with liver synthetic dysfunction due to decompensated cirrhosis. Taken together, Algorithms 1 and 3 can be used to reliably identify intrahepatic cholangiocarcinoma cases and Algorithms 4, 5, 6, and 8 can be used to reliably identify extrahepatic cholangiocarcinoma within VA data in future analyses.

Our study has several potential limitations. First, there was the potential for misclassification of cholangiocarcinoma and subtype during adjudication. Indeed, the lower kappa statistics observed for Algorithms 4, 6, and 8 in Table 4 reflected the clinical challenges in ascertaining the precise location of perihilar (Klatskin) and distal cholangiocarcinoma. We tried to minimize this by using pre‐specified case definitions for both cholangiocarcinoma and subtype and employing two independent reviewers with a third to arbitrate disagreements. Second, we did not determine the negative predictive value, sensitivity, or specificity of our algorithms, since a registry of all confirmed cholangiocarcinoma cases does not exist within the VA. However, since cholangiocarcinoma is a rare malignancy, the negative predictive value of our algorithms was expected to be very high. Moreover, algorithms with high specificity are ideal for identifying rare events [20]. However, our decision to prioritize specificity and PPV might result in an underestimation of cholangiocarcinoma incidence. Third, our algorithms may not be transportable to other data sources and should be evaluated prior to use outside the VA. Fourth, we did not develop algorithms based on ICD‐9/10 diagnosis codes, but this should be considered in future studies to increase the ability to identify more cases, since there is a time lag between the diagnosis and entry into the cancer registry.

Our study had a number of strengths. We developed and applied rigorous case definitions to classify definite and probable cholangiocarcinoma diagnoses and subtypes. We employed two hepatologists to independently adjudicate cholangiocarcinoma and its location and had a third hepatologist arbitrate any disagreements. Moreover, we explored various ICD‐O‐3 histology codes and evaluated the potential usefulness of the adenocarcinoma NOS ICD‐O‐3 histology code for identifying cholangiocarcinoma. Finally, we incorporated maximum Tbili level into two algorithms to improve the accuracy of identifying intrahepatic cholangiocarcinoma from extrahepatic cholangiocarcinoma.

In conclusion, we developed eight ICD‐O based algorithms to identify cholangiocarcinoma events and ascertain cholangiocarcinoma subtype within national VA data with high PPV. These algorithms could be used in future studies within the VA to evaluate the determinants and outcomes of cholangiocarcinoma and its locations.

### 4.1 Plain Language Summary

There are major knowledge gaps on the determinants and comparative effectiveness of medical therapies for cholangiocarcinoma and its subtypes, largely because methods to validly identify this malignancy within real‐world data have been lacking. We developed and evaluated the performance of eight case‐finding algorithms for cholangiocarcinoma and its subtypes using cancer registry coded diagnoses, alone or in combination with total bilirubin values, within electronic healthcare data of the US Veterans Health Administration. All eight algorithms had a positive predictive value (PPV) of at least 83.8% (range: 83.8%–100.0%) for hepatologist‐confirmed definite or probable cholangiocarcinoma. Two of the three algorithms created to identify intrahepatic cholangiocarcinoma had high PPV for definite or probable intrahepatic cholangiocarcinoma, ranging from 88.8%–91.3%. Four of the five algorithms created to identify extrahepatic cholangiocarcinoma had high PPV for definite or probable extrahepatic cholangiocarcinoma, ranging from 80.0%–94.0%. These algorithms could be used in future pharmacoepidemiologic studies to evaluate medications associated with intrahepatic or extrahepatic cholangiocarcinoma within US Veterans Health Administration data.

## Conflicts of Interest

The authors declare no conflicts of interest.

## Supporting information


**Table S1.** Cholangiocarcinoma‐related ICD‐O‐3 histology and ICD‐O‐3 topography codes and descriptions.
