# Supplementary material for: Validity of Diagnostic Codes and Laboratory Tests to Identify Cholangiocarcinoma and Its Subtypes
Source: Pharmacoepidemiol Drug Saf. 2025 May 6;34(5):e70154. doi: 10.1002/pds.70154 (PMC12055315; doi:10.1002/pds.70154)
Supplement: Supplementary file 1 — Table S1. Cholangiocarcinoma‐related ICD‐O‐3 histology and ICD‐O‐3 topography codes and descriptions. [file PDS-34-e70154-s001.docx]

**Supplementary Table 1. Cholangiocarcinoma-related ICD-O-3 histology and ICD-O-3 topography codes and descriptions.**

| **ICD-O-3 Histology Codes** | | **ICD-O-3 Topography Codes** | |
| --- | --- | --- | --- |
| **ICD-O-3 code** | **Description** | **ICD-O-3 code** | **Description** |
| 8160* | Cholangiocarcinoma | C22.0 | Liver |
| 8161* | Bile duct cystadenocarcinoma | C22.1 | Intrahepatic bile duct |
| 8162* | Klatskins tumor | C24.0 | Extrahepatic bile duct |
| 8010** | Carcinoma NOS | C23.9 | Gallbladder |
| 8020** | Carcinoma undifferentiated type | C24.9 | Biliary tract NOS |
| 8140** | Adenocarcinoma NOS |  |  |
| 8144** | Adenocarcinoma intestinal type |  |  |
| 8260** | Papillary adenocarcinoma |  |  |
| 8262** | Villous adenocarcinoma |  |  |
| 8480** | Mucinous adenocarcinoma |  |  |
| 8481** | Mucin producing adenocarcinoma |  |  |
| 8490** | Signet ring cell carcinoma |  |  |
| 8500** | Infiltrating duct carcinoma |  |  |
| 8560** | Adenosquamous carcinoma |  |  |
| 8070** | Squamous cell carcinoma |  |  |

Abbreviations: ICD-O-3=International Classification of Diseases for Oncology, Third Edition; NOS=Not otherwise specified.

*8160, 8161, 8162: ICD-0-3 histology codes specific for cholangiocarcinoma.

**8010, 8020, 8140, 8144, 8260, 8262, 8480, 8481, 8490, 8500, 8560, 8070: Other possible ICD-0-3 codes for cholangiocarcinoma.
